# Quantifying and characterizing hourly human exposure to malaria vectors bites to address residual malaria transmission during dry and rainy seasons in rural Southwest Burkina Faso

**DOI:** 10.1186/s12889-021-10304-y

**Published:** 2021-01-30

**Authors:** D. D. Soma, B. Zogo, P. Taconet, A. Somé, S. Coulibaly, L. Baba-Moussa, G. A. Ouédraogo, A. Koffi, C. Pennetier, K. R. Dabiré, N. Moiroux

**Affiliations:** 1 Institut de Recherche en Sciences de la Santé (IRSS), Bobo-Dioulasso, Burkina Faso; 2 Université Nazi Boni (UNB), Bobo-Dioulasso, Burkina Faso; 3 MIVEGEC, Univ. Montpellier, CNRS, IRD, Montpellier, France; 4 Institut Pierre Richet (IPR), Bouaké, Côte d’Ivoire; 5 Université d’Abomey Calavi, Abomey-Calavi, Benin

**Keywords:** Diébougou, LLIN, *Anopheles*, Humans, Behaviours, Residual transmission

## Abstract

**Background:**

To sustain the efficacy of malaria vector control, the World Health Organization (WHO) recommends the combination of effective tools. Before designing and implementing additional strategies in any setting, it is critical to monitor or predict when and where transmission occurs. However, to date, very few studies have quantified the behavioural interactions between humans and *Anopheles* vectors in Africa. Here, we characterized residual transmission in a rural area of Burkina Faso where long lasting insecticidal nets (LLIN) are widely used.

**Methods:**

We analysed data on both human and malaria vectors behaviours from 27 villages to measure hourly human exposure to vector bites in dry and rainy seasons using a mathematical model. We estimated the protective efficacy of LLINs and characterised where (indoors vs. outdoors) and when both LLIN users and non-users were exposed to vector bites.

**Results:**

The percentage of the population who declared sleeping under a LLIN the previous night was very high regardless of the season, with an average LLIN use ranging from 92.43 to 99.89%. The use of LLIN provided > 80% protection against exposure to vector bites. The proportion of exposure for LLIN users was 29–57% after 05:00 and 0.05–12% before 20:00. More than 80% of exposure occurred indoors for LLIN users and the estimate reached 90% for children under 5 years old in the dry cold season.

**Conclusions:**

LLINs are predicted to provide considerable protection against exposure to malaria vector bites in the rural area of Diébougou. Nevertheless, LLIN users are still exposed to vector bites which occurred mostly indoors in late morning. Therefore, complementary strategies targeting indoor biting vectors in combination with LLIN are expected to be the most efficient to control residual malaria transmission in this area.

**Supplementary Information:**

The online version contains supplementary material available at 10.1186/s12889-021-10304-y.

## Background

Massive distribution of long-lasting insecticidal nets (LLINs) is a core intervention for malaria control in Burkina Faso. Scaling-up of coverage with LLIN in sub-Saharan Africa has been very successful between 2000 and 2015 during which malaria morbidity and mortality have dropped considerably [1]. Unfortunately, this significant progress is stalling or even reversing in some countries. Burkina Faso is indeed one of the sixteen (16) in the world that documented an increase in malaria burden from 2016 to 2017 [2]. This trend might be attributed to the recent increases in prevalence and strength of pyrethroid resistance in malaria vectors [3–5]. Another possible cause is the development of behavioural resistance in vector populations [6–8]. In sub-Saharan Africa, there have been many reports of changes in vector species and/or vector biting behaviours to avoid contact with LLIN [6–8]. Such changes in vector populations are considered by many specialists as an important threat for indoor control strategies such as LLIN [9, 10].

To sustain the efficacy of vector control, the WHO recommends the combination of effective tools [11]. At present, there are a number of recommended tools available and many others under development that can potentially be combined with LLIN [12, 13]. However, national malaria control programs (NMCPs) are now facing challenges to design effective control strategies due to high variations in malaria epidemiology between and even within countries [14]. To do so, NMCP must be able to monitor or predict when and where transmission occurs and to characterize residual transmission (i.e. the transmission that escapes vector control by LLINs).

In order to compare the impact of LLINs on human exposure to malaria vectors bite among sites, Killeen et al. [15] developed an approach that quantify behavioural interactions between mosquitoes and humans. The approach use measures of indoor and outdoor vector biting as well as the distribution of people outdoors, indoors and under LLINs for each hour of the night. It produces average hourly and nightly weighted estimates of exposure occurring indoors and outdoors as well as estimates of prevented exposure. The analytical model developed by Killeen et al. and extended by Geissbühler et al., [16] is therefore a useful tool to estimate protective efficacy of LLINs and to characterize residual transmission. Indeed, it allows to identify where (indoors vs. outdoors) and during which hours LLIN users are exposed to anopheles bite, i.e. where and when residual transmission is expected to occur. Numerous studies have used this model in Africa [15–30]. However until now, only one of these studies has reported exposure estimates for sites located in Burkina Faso [18].

The present study aims to provide and discuss up-to-date estimates of human exposure to *Anopheles* bite and to characterise residual malaria transmission in an area of Burkina Faso where malaria vectors shows high levels of pyrethroid resistance [31]. Results of entomological surveys previously reported [31] were used in combination with human behavioural data to quantify, through the Killeen’s model, the behavioural interactions between humans and *Anopheles* mosquitoes during both dry and rainy seasons in the Diébougou area, southwest Burkina Faso. Data were collected during the pre-intervention stage of a large randomized control trial designed to investigate whether the combination of LLINs with other vector control tools can provide additional protection over malaria cases and transmission.

## Methods

**Fig. 1:**
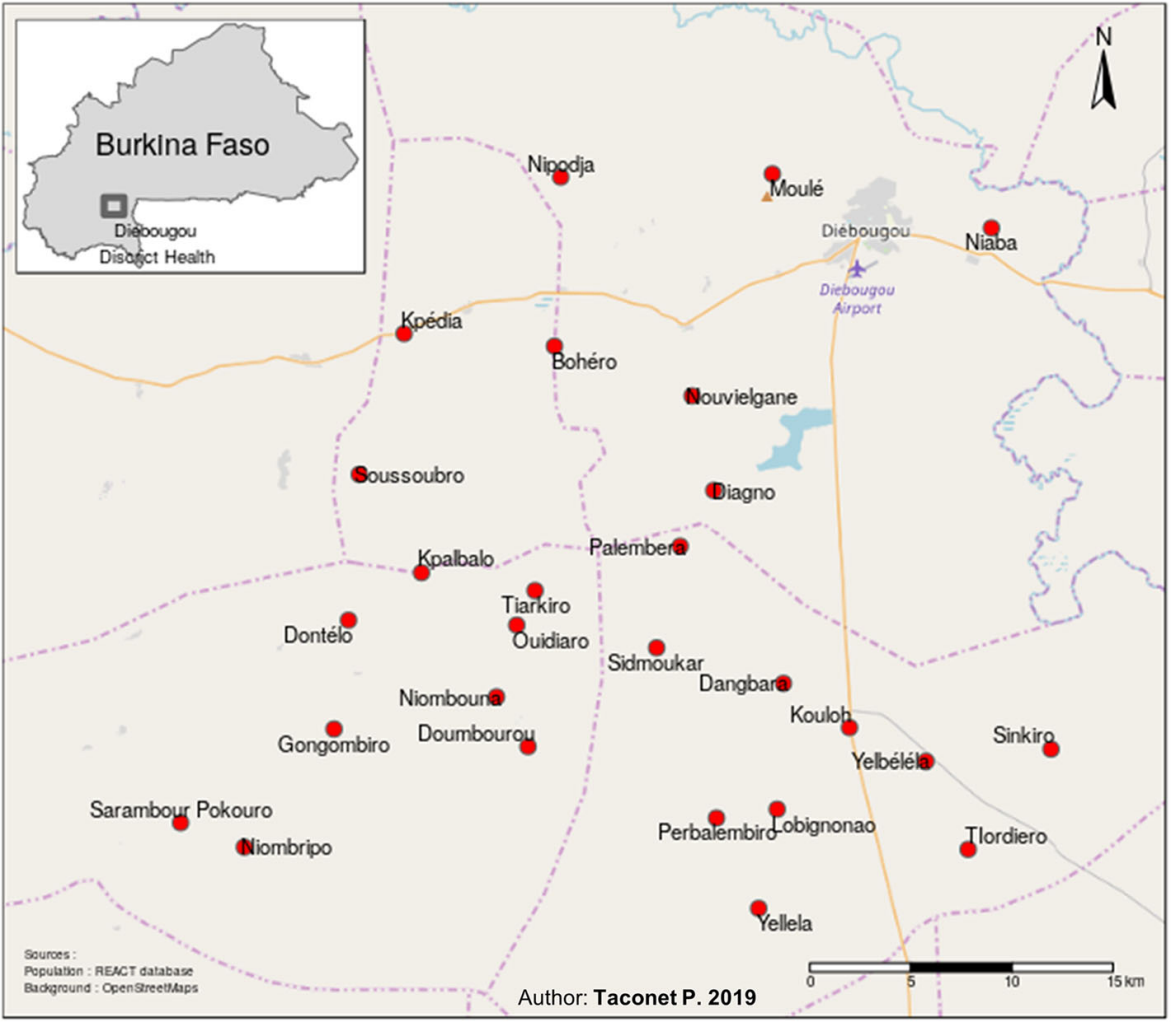
Map of the study area and villages surveyed. *Background of the map was produced with open data (under ODbL Licence) from* openstreetmap.org*. Data of village locations are own and obtained through the REACT project*

This study was conducted in 27 villages located in the Diébougou health district, southwest Burkina Faso (Fig. 1). These villages were selected based on geographical (distance between two villages higher than 2 km and accessibility during the rainy season) and demographic (a population size ranging from 200 to 500 inhabitants) criteria [31] to participate in a randomized controlled trial. The climate in the study area is tropical with one dry season from October to April (including a cold period from December to February and a hot period from March to April) and one rainy season from May to September. Average daily temperature amplitudes are 18–36 °C, 25–39 °C and 23–33 °C in dry cold, dry hot and rainy season, respectively. The mean annual rainfall is 1200 mm. The natural vegetation is dominated by wooded savannah dotted with clear forest gallery. The main economic activity is agriculture (cotton growing and cereals) followed by artisanal gold mining and production of coal and wood [32, 33]. In the study area as in the whole country, a mass distribution of LLINs (PermaNet 2.0) was carried out by the NMCP in July 2016. No LLINs were distributed by our teams.

**Fig. 2:**
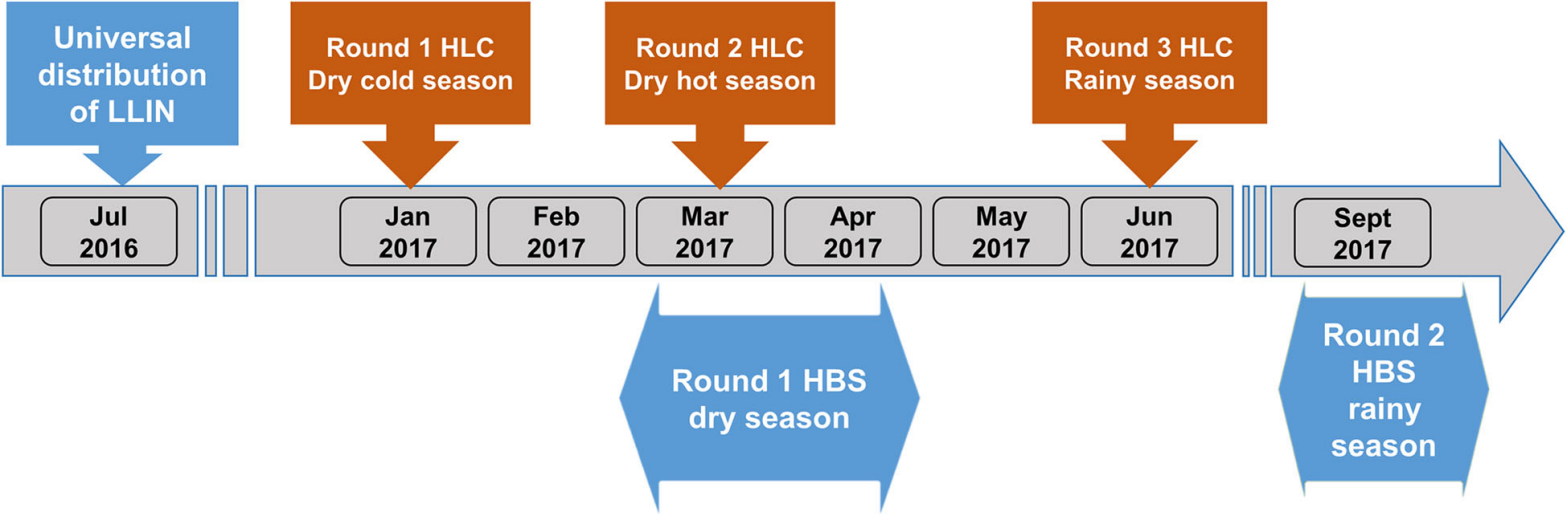
Timeline of long-lasting insecticidal impregnated net (LLIN) distribution campaigns, human behaviours surveys (HBS), and entomological surveys. *HLC: Human Landing Collection*

The study involved the conduct of three entomological surveys and two human behavioural surveys. Figure 2 shows the timeline of the study. We conducted three entomological surveys in the dry cold (January 2017), dry hot (March 2017) and rainy seasons (June 2017). During each survey, we collected mosquitoes using the standard method of human landing catch (HLC). Mosquitoes were sampled both indoors and outdoors from 17:00 h to 09:00 h in 4 houses per villages during one night [31]. In each study village, two teams of eight collectors were deployed, with the first team collecting from 17:00 h to 01:00 h and the second from 01:00 h to 09:00 h. All the collected mosquitoes were morphologically identified [34, 35] and *Anopheles spp.* mosquitoes were subsequently identified to the species level by polymerase chain reaction [36–38]. Detailed descriptions of the methods used are provided in our previous publication [31]. In the current work, we aggregate data for all species belonging to the *Anopheles* genus (*Anopheles spp*) in order to have appropriate data regarding malaria vectors behaviour. Overall, *Anopheles funestus s.s* was the main malaria vector in the study area during the dry cold season [31]. During the dry hot and rainy seasons, *Anopheles coluzzii* and *Anopheles gambiae s.s* were the dominant species. The mean endophagy rate (ER) of malaria vectors was 63.23, 50.18 and 57.18% during the dry cold, dry hot and rainy seasons, respectively [31].

In order to obtain appropriate data regarding relevant human behaviours, we surveyed 401 and 339 randomly selected households in dry (end of February to April 2017) and rainy (September 2017) seasons, respectively (corresponding to an average of 15 and 13 households per village). Among people usually leaving in each selected household, we randomly selected 3 persons (maximum) belonging to each of the 3 following age groups: 0–5 years old, 6–17 years old and ≥ 18 years old. We asked the head of the household the time at which each selected person (1) entered and exited his own house the night preceding the survey and (2) the time each LLIN user entered and exited his sleeping space the night preceding the survey (the questionnaire was previously published in supplementary Text S1 of [20]). In order to know the relative weight of each age group in the population, we recorded the number of individuals belonging to these groups in each household. A total of 3045 and 2880 individuals were surveyed in dry and rainy seasons, respectively, representing 35.08 and 33.17% of the 27 villages’ population according to a census carried out by our team in 2016 [31]. The human behavioural surveys were carried out in the same villages where mosquitoes were collected. The selections of households for human behavioural surveys and of houses for entomological survey were independent. Data were recorded using tablets running Open Data Kit (ODK) forms.

From data of each entomological survey, we calculated indoor and outdoor hourly biting rates (i.e. the number of *Anopheles* mosquitoes collected per human per hour) at the village level and for the whole study area. At the same scales, we calculated from data of each human behavioural survey the hourly proportions of people being indoors or under an LLIN. Hourly biting rates and hourly distribution of people were combined to calculate estimates of human exposure to *Anopheles spp.* bites in the dry season (both cold and hot) and the rainy season using an extension of the Killeen’s model [15] as previously described in Geissbühler et al. [16] and Moiroux et al. [20] and detailed in Additional file 1.

Since only one survey of human behaviour was carried out in dry season, we used the same human behaviour data to model human exposure to *Anopheles* bite during both dry cold and dry hot seasons.

We estimated the average *true* personal protection (P*) of using an LLIN (i.e. the proportion of exposure to all bites occurring both indoors and outdoors that is prevented by using an LLIN) as well as the proportion of exposure which occurred indoors for LLIN users either accounting for the personal protection provided by net use (*π*_*i,n*_) or ignoring it to compare with available estimates for unprotected people (*π*_*i*_) [16] (Additional File 1). Exposure when sleeping under an LLIN was assumed to be reduced by 92% [20]. Moreover, to characterize residual transmission, we calculated the proportion of exposure occurring before 20:00 (*π*_*e,n*_) and after 5:00 (*π*_*m,n*_) that are the times respectively preceding and following the period when most (> 50%) of LLIN users are protected (Additional File 1).

All the exposure estimates (i.e. P*, *π*_*i,n*_, *π*_*i*_, *π*_*e,n*_, *π*_*m,n*_) were calculated at the village and study area levels, for each age group as well as for the whole population. The relative weight of each age classes in the population was taken into account when calculating exposure values at the population level (see Additional File 1). For these calculation and to produce figures, we developed an R [39] package named “biteExp” (https://github.com/Nmoiroux/biteExp).

## Results

**Fig. 3:**
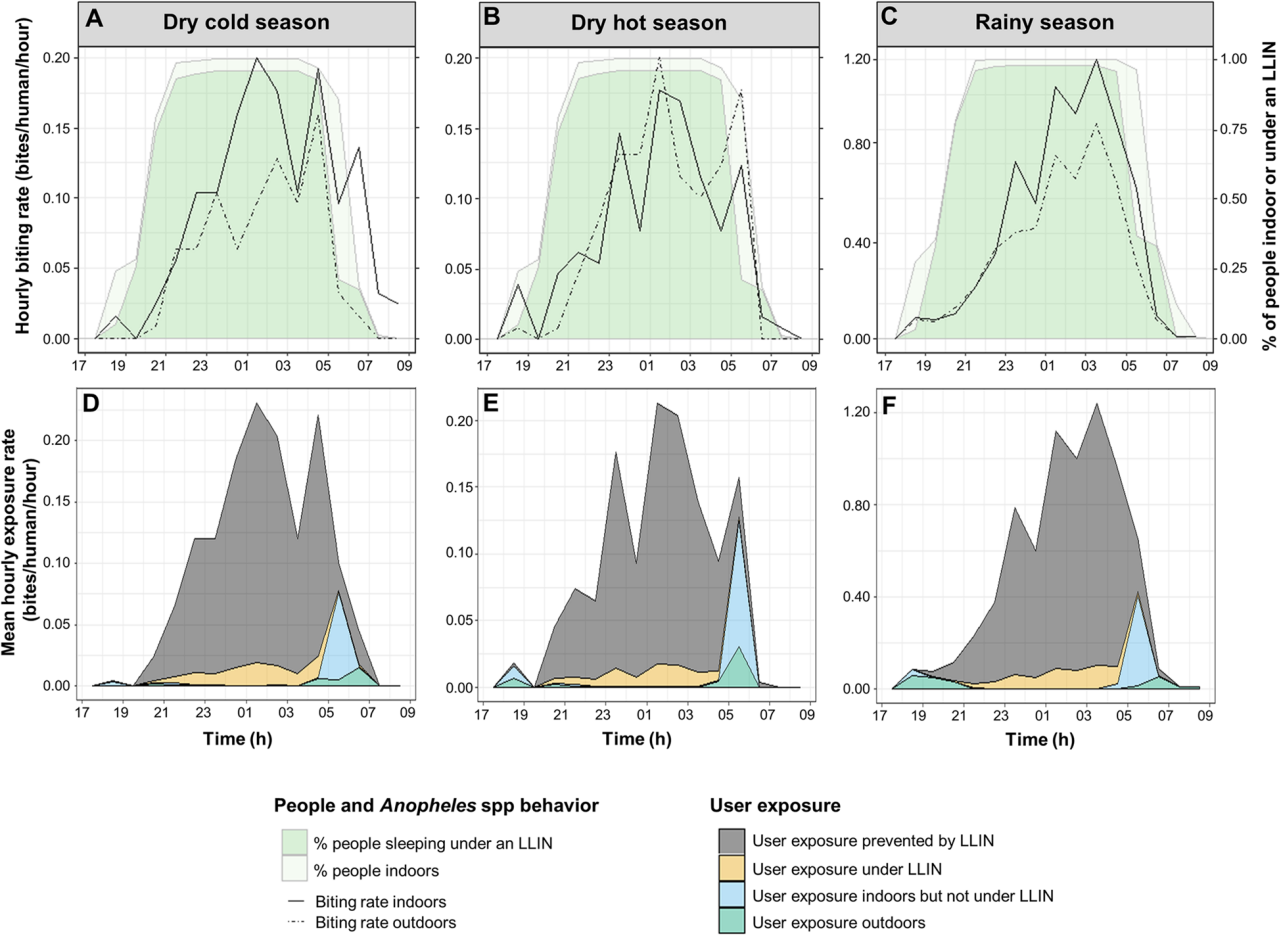
Hourly human and *Anopheles* spp. behavior (**a, b, c**) and hourly exposure to bites of LLIN users (**d, e, f**), Burkina Faso. *Human behavioural data in panel A and B are the same (only one dry season survey) but plotted with different entomological data*

The average declared LLIN use rate was very high in the study population ranging from 95.49% in the dry season to 99.67% in the rainy season (Table 1). The declared LLIN use rate was higher in the 0–5 years old age group (97.87% in the dry season to 100% in the rainy season) compared to children aged 6–17 years old (95.36% in the dry season to 99.79% in the rainy season) and adults (92.45% in the dry season to 99.19% in the rainy season) (Table 1). However, we found that the LLIN use rate varied among villages (see Additional file 2) with the lowest rates observed in Kpédia (68.42%), Palembera (71.73%) and Diagnon (78.78%) in the adults group during the dry season. In the other villages LLIN use rates ranged from 80 to 100% whatever the season (see Additional file 2). Figure 3 shows humans and *Anopheles* behaviour profiles as well as average hourly exposure and prevented exposure to bites for LLIN users in our study area.

**Table 1:** Average LLIN use rates, true average protection efficacy of LLINs against exposure to vector bites and proportions of indoors, “before bed” and “after bed” exposure to *Anopheles* bites for both LLIN users and non-users in 27 villages of the Diébougou area, Burkina Faso

Season	Age (years)	LLIN use rate (%[min-max])	^a^True average LLIN personal protection efficacy (% [min-max])	Exposure indoors (%[min-max])		Exposure before 20:00 h (%[min-max])		Exposure after 05:00 h (%[min-max])	
				LLIN users	Non-users	LLIN users	Non-users	LLIN users	Non-users
**Dry cold season**	**18+**	92.45 [68–100]	83.44 [0–92]	79.92 [0–100]	96.67 [0–100]	0.07 [0–0.13]	0.04 [0–0.34]	44.99 [0–100]	8.16 [0–100]
	**6 to 17**	95.36 [71–100]	83.79 [0–92]	85.44 [0–100]	97.64 [0–100]	0.58 [0–1]	0.12 [0–0.73]	48.93 [0–100]	9.01 [0–100]
	**0 to 5**	97.87 [81–100]	86.73 [0–92]	90.52 [0–100]	98.74 [0–100]	3.93 [0–100]	0.62 [0–100]	40.23 [0–100]	12.20 [0–100]
	**population**	95.49 [77–100]	84.93 [0–92]	85.62 [0–100]	97.83 [0–100]	1.66 [0–100]	0.31 [0–100]	44.50 [0–100]	10.11 [0–100]
**Dry hot season**	**18+**	92.45 [68–100]	78.00 [0–92]	69.57 [19–100]	93.31 [75–100]	3.38 [0–26]	0.82 [0–1]	57.20 [0–100]	13.19 [0–100]
	**6 to 17**	95.36 [71–100]	79.88 [2–92]	82.70 [21–100]	96.52 [72–100]	4.57 [0–5]	0.99 [0–2]	56.20 [0–100]	12.27 [0–100]
	**0 to 5**	97.87 [81–100]	83.63 [13–92]	88.73 [29–100]	98.15 [82–100]	11.30 [0–20]	2.13 [0–3]	43.95 [0–100]	12.32 [0–100]
	**population**	95.49 [77–100]	80.89 [5–92]	80.54 [24–100]	96.28 [78–100]	6.56 [0–30]	1.41 [0–2]	52.19 [0–100]	12.55 [0–100]
**Rainy** **season**	**18+**	99.19 [92–100]	79.13 [53–92]	75.61 [11–100]	94.91 [62–100]	10.08 [0–23]	2.17 [0–5]	42.90 [0–90]	9.81 [0–44]
	**6 to 17**	99.79 [94–100]	81.83 [51–92]	83.28 [45–100]	96.96 [91–100]	10.24 [0–25]	2.22 [0–8]	48.59 [0–91]	10.42 [0–50]
	**0 to 5**	100.00	87.00 [72–92]	89.21 [69–100]	98.60 [96–100]	11.33 [0–19]	2.31 [0–11]	33.88 [0–85]	10.55 [0–50]
	**population**	99.67 [97–100]	82.82 [58–92]	81.93 [27–100]	96.90 [82–100]	10.47 [0–23]	2.23 [0–9]	42.40 [0–89]	10.27 [0–48]

Min and max reported in brackets give the value recorded in the village with the lower and the higher average value, respectively

^a^True average LLIN personal protection efficacy: estimated proportion of *Anopheles* bites prevented by the use of a LLIN

The majority of the population was indoors from 20:00 in both dry and rainy seasons (Fig. 3a, b and c). These populations woke up around 05:00 in the early morning in all seasons (Fig. 3a, b and c). Most of the total exposure to *Anopheles* bites occurred indoors (> 94% for non-users, Table 1) but was largely preventable by using of LLIN (Fig. 3d, e and f). Indeed, LLIN were estimated to provide average ‘true’ personal protection against 84.93, 80.89 and 82.82% of exposure in dry cold season, dry hot season and rainy season, respectively (Table 1, Additional file 3). The peak of exposure for users occurred indoors between 05:00 and 06:00 just before sunrise whatever the season (Fig. 3d, e and f). On average, between 33 and 57% of residual exposure of LLIN users occurred after wake up (after 5:00) depending on age groups. Early bites (before 20:00) represented less than 12% of the residual exposure of LLIN users (Table 1).

## Discussion

The average declared LLIN use rate was very high (> 95%) in all age groups of our study population. The LLIN use rate was slightly higher in children under five years of age than the rest of the population. This finding is consistent with results from a multi-country analysis that revealed that the most vulnerable groups are preferentially protected by LLIN in sub-Saharan Africa [40]. At the village level, the use rate rarely fall under 80%, being consistently higher than the nationwide LLIN use value of 67% published by WHO in 2017 [41]. This may be explained by the fact that the study was conducted approximately 6 months after a wide LLIN distribution. However, our reported LLIN use may be overestimated because it was based on self-reported survey questions, the most commonly used method to assess bednet use [42]. To more accurately estimate LLIN use, future studies quantifying human exposure to mosquito bites should consider using other measurement methods such as electronic monitoring devices [43, 44].

This study shows that the overall protective efficacy of LLINs against vector bites in the rural area of Diébougou was high (80–85%) during the three seasons. Our estimates for LLIN personal efficacy were comparable with those found in Benin (80 and 87%) [20] but were higher than those reported elsewhere such as in Kenya (51%) [21] and Tanzania (70, 59 and 38%) [15, 16]. Our results support strongly the use of LLIN as a primary malaria vector control tool in the area. Nevertheless, such a protection level (85% in average) has to be put into perspective with the high malaria transmission and endemicity [31] in order to measure/realize the importance of malaria residual transmission in the area.

We estimated that 33–57% of residual exposure to *Anopheles* bites of LLIN users occurred after 5:00 and 0.07–12% occurred before 20:00 when most of users are awake. The proportion of exposure for LLIN users has been higher in the late part of the morning than in the early part of the evening in some settings while the opposite trend has been observed in other settings [15, 20, 23, 45]. In our study area, over 80% of human exposure to vector bites occurred indoors for LLIN users. For children under 5 years who use LLINs, the exposure rate occurring indoors reached 90%. Therefore, these results suggest that adding other indoor intervention such as indoor residual spraying (IRS) to LLINs would be relevant to reduce malaria transmission in the rural area of Diébougou. In 2017, 28 countries in the world have implemented IRS in combination with LLINs to combat malaria [2]. IRS contributed to an estimated 10 (5–14)% of the reduction in malaria burden achieved recently [1]. When used together, IRS and LLINs are expected to target vectors at different stages of their gonotrophic cycle using insecticides with different mode of action. However, trials assessing the impact of the combination IRS + LLIN over LLIN use alone have yielded conflicting results [46–51]. House improvement is another indoor measure which needs careful consideration and deep investigations. Indeed, house improvement has been strongly associated with reduced malaria transmission and disease in many studies [52–54]. The main house improvement interventions studied are closed eaves, closed ceilings, window screens and metal-roof houses as opposed to eaves, ceilings, windows openings and thatched-roof houses. Such improvements protect against malaria by providing physical barriers that prevent vectors from entering houses and can reduce vector survivorship [52, 55]. Nonetheless, there is compelling evidence that even a full coverage of effective measures within houses would not be sufficient to suppress transmission of malaria in Africa [56].

In this study, we evidenced that a significant proportion of LLIN users exposure to vector bites occurred outdoors (ranging from 9.48 to 30.43%), with the highest estimate recorded in adults (over the age of 18 years old) during the dry hot season. Many studies conducted in various areas of Africa reported similar or even higher estimates of exposure occurring outdoors [15, 16, 18, 45]. Recently, a systematic review categorized Burkina Faso along with Eritrea, Ethiopia, Gabon, and Tanzania as countries with high levels of outdoor vector biting [10]. However, our results do not fully support this categorization since we show that both LLIN users and LLIN non users are far more exposed to vector bites indoors than outdoors in the study area. Nevertheless, strategies targeting outdoor bites would probably be required to achieve malaria elimination in the area.

Almost all the existing indoor vector control strategies face two important evolutive challenges. First, they induce a strong selective pressure on physiological resistance in vector populations because they almost all rely on synthetic chemicals [57]. Second, they also induced selective pressure for behavioral changes in vector populations resulting in a reduced contact with interventions [57]. In this context, there is a crucial need to monitor these resistance mechanisms, as well as residual transmission, after the deployment of strategies to inform decision makers in order to allow them to adapt their strategic plans.

## Conclusions

This study showed that the use of LLINs prevented more than 80% of *Anopheles* bite exposure. Nevertheless, LLIN users are still exposed to vector bites which occurred mostly indoors in late morning. Therefore, complementary strategies targeting indoor biting vectors in combination with LLIN are expected to be the most efficient to control residual malaria transmission in this area.

## Supplementary Information

**Additional file 1 **Model specification. Formulae used to calculate mean exposure to bite, true average personal protection efficacy of LLINs (P*), proportions of indoor (π_*i*_ *and π*_*i,n*_*), “before bed” (π*_*e*_ *and π*_*e,n*_*) and “after bed” (π*_*m*_ *and π*_*m,n*_*) exposure to bite.*

**Additional file 2 **LLIN Use rate per village. *N: number.*

**Additional file 3 **True average protection efficacy of LLINs against transmission and Proportions of indoors, early evening and late morning exposure to *Anopheles* bites per village. *NA: Not Applicable.*

## Data Availability

The datasets used and/or analyzed during the current study are available from the corresponding author on reasonable request.

## Abbreviations

HBS: Human behaviours surveys
HLC: Human landing catch
ER: Endophagy Rate
IRS: Indoor Residual Spraying
LLIN: Long-Lasting Insecticidal Nets
ODK: Open Data Kit
NMCP: National Malaria Control Programs
WHO: World Health Organization
