# Supplementary material for: Quantifying and characterizing hourly human exposure to malaria vectors bites to address residual malaria transmission during dry and rainy seasons in rural Southwest Burkina Faso
Source: BMC Public Health. 2021 Jan 30;21:251. doi: 10.1186/s12889-021-10304-y (PMC7847557; doi:10.1186/s12889-021-10304-y)
Supplement: Supplementary file 1 — Additional file 1 Model specification. Formulae used to calculate mean exposure to bite, true average personal protection efficacy of LLINs (P*), proportions of indoor (πi and πi,n), “before bed” (πe and πe,n) and “after bed” (πm and πm,n) exposure to bite. [file 12889_2021_10304_MOESM1_ESM.docx]

### Formulae used to calculate mean exposure to bite, true average personal protection efficacy of LLINs (P*), proportions of indoor exposure to bite (π_i_ and π_i,n_), and proportions of “before bed” (π_e_ and π_e,n_) and “after bed” (π_m_ and π_m,n_):

Data collected from LLIN users during the human behavioural survey were used to calculate the proportions of people declaring being indoors (*I_t_*) and the proportion of people declaring being protected by an LLIN (*S_t_*) for each surveyed hour of the night (*t*), which included 16 hours between 17:00 and 9:00, which were numbered from 5 to 20. These data were used to calculate the mean biting rate experienced by an unprotected person and a protected net user (*n*).

The **mean biting rate experienced by a non-user (*B_t_*)** for each surveyed time of the night (t) can be calculated as the proportion of people declaring being indoors (*I_t_*) multiplied by the indoor biting rate at that time (*B_i,t_*) plus the proportion of people declaring being outdoors (*1- I_t_*) multiplied by the outdoor biting rate at that time (*B_o,t_*):

$B_{t}=B_{i,t}I_{t}+B_{o,t}(1-I_{t})$ (1)

Then **the mean biting rate (*B*) experienced by a non-user** between 17:00 and 9:00 can be calculated by summing hourly biting rates as follow:

$B_{5\to20}=\sum_{t=5}^{20} B_{t}$ (2)

The **mean biting rate experienced by a LLIN user (*B_n_*)** between 17:00 and 9:00 was calculated by adjusting the indoor biting rate for the protected fraction (*S_t_*) of the population in proportion to the personal protection (*P*) provided by LLINs Permanet 2.0™ (i.e. the LLINs distributed in the villages of the study) [1, 2]:

$B_{n,5\to20}=\sum_{t=5}^{20} B_{n,t}=\sum_{t=5}^{20} \left[ B_{i,t}{((I}_{t}{-S}_{t})+{S_{t}(1-P))+B}_{o,t}(1-I_{t}) \right]$ (3)

The personal protection (*P*) was set to 0.92 according to experimental hut trials of Permanet 2.0™ in Benin in an area (Malanville) with very low levels of pyrethroid resistance [3].

The ***true* average personal protection efficacy of a LLIN (*P**)** against exposure to bites was calculated as the overall reduction of biting rate for LLIN users (compared to non-users) [1] using the following formula:

$P^{*}=1-({B_{n, 5\to20}}/{B_{5\to20}})$ (4)

The **proportion of bites experienced indoors by a non-user (*π_i_*)** was calculated by comparing the mean biting rates experienced indoors to the overall mean biting rate:

$\pi_{i}=\sum_{t=5}^{20} \left[ B_{i, t}I_{t} \right]/{B_{5\to20}}$ (5)

The **proportion of bites experienced indoors by an LLIN user (*π_i,n_*)** was calculated by comparing the mean biting rates experienced indoors to the overall mean biting rate:

$\pi_{i,n}=\sum_{t=5}^{20} \left[ B_{i,t}{((I}_{t}{-S}_{t})+S_{t}(1-P) \right]/{B_{n,5\to20}}$ (6)

The **proportion of bites experienced after 5:00 in the morning by a non-user (*π_m_*)** was calculated by comparing the mean biting rate experienced between 5:00 and 9:00 to the overall mean biting rate (i.e. between 17:00 and 9:00):

$\pi_{m}={B_{17\to20}}/{B_{5\to20}}$ (7)

The **proportion of bites experienced after 5:00 by an LLIN user (*π_m,n_*)** during was calculated by comparing the mean biting rate experienced between 5:00 and 9:00 to the overall mean biting rate (i.e. between 17:00 and 9:00):

$\pi_{m,n}={B_{n,17\to20}}/{B_{n,5\to20}}$ (8)

The **proportion of bites experienced before 20:00 in the evening by a non-user (*π_e_*)** was calculated by comparing the mean biting rate experienced between 17:00 and 20:00 to the overall mean biting rate (i.e. between 17:00 and 9:00):

$\pi_{e}={B_{5\to7}}/{B_{5\to20}}$ (9)

The **proportion of bites experienced after 20:00 by an LLIN user (*π_e,n_*)** during was calculated by comparing the mean biting rate experienced between 17:00 and 20:00 to the overall mean biting rate (i.e. between 17:00 and 9:00):

$\pi_{e,n}={B_{n,5\to7}}/{B_{n,5\to20}}$ (10)

Proportions of people of each age classes declaring being indoors (*I_t,a_*) and declaring being protected by an LLIN (*S_t,a_*) for each surveyed hour of the night (*t*) were weighted by their relative proportion in the population (*P_a_*). The resulting weighted proportion were summed together to give (*I_t,p_*) and (*S_t,p_*) at the population (*p*) scale. We numbered age classes (*a*) from 1 to 3 corresponding to age 0-5, 6-17 and >18, respectively.

$I_{t,p}=\sum_{a=1}^{3} I_{t,a}P_{a}$ (11)

$S_{t,p}=\sum_{a=1}^{3} S_{t,a}P_{a}$ (12)

(*I_t,p_*) and (*S_t,p_*) were used in expressions (1) to (10) to obtain estimates at the population scale.

# References

1. Killeen GF, Kihonda J, Lyimo E, et al. Quantifying behavioural interactions between humans and mosquitoes: evaluating the protective efficacy of insecticidal nets against malaria transmission in rural Tanzania. BMC Infect Dis 2006;6:161

2. Geissbühler Y, Chaki P, Emidi B, Govella NJ, Shirima R, Mayagaya V, et al. Interdependence of domestic malaria prevention measures and mosquito-human interactions in urban Dar es Salaam, Tanzania. Malar J. 2007;6:126.

3. Corbel V, Chabi J, Dabire RK, et al. Field efficacy of a new mosaic long-lasting mosquito net (PermaNet 3.0) against pyrethroid-resistant malaria vectors: a multi centre study in Western and Central Africa. Malar J 2010;9:113
